# Onion-Peel Carbon Quantum Dots: Antimicrobial Effect and Biofilm Control on Food Contact Surfaces

**DOI:** 10.3390/foods14244296

**Published:** 2025-12-13

**Authors:** Ji Min Ahn, Yeon Ho Kim, Jong-Whan Rhim, Ki Sun Yoon

**Affiliations:** Department of Food and Nutrition, College of Human Ecology, Kyung Hee University, 26 Kyungheedae-ro, Dongdaemun-gu, Seoul 02447, Republic of Korea; ajm929@khu.ac.kr (J.M.A.); youngheum91@naver.com (Y.H.K.); jwrhim@khu.ac.kr (J.-W.R.)

**Keywords:** onion peel, carbon quantum dots, biofilm, *Salmonella typhimurium*, *Listeria monocytogenes*

## Abstract

As by-products rich in flavonoids and phenolic compounds, onion peels are globally undervalued and often discarded. This study reports the synthesis of carbon quantum dots (CQDs) from onion peels and evaluates their antimicrobial effectiveness against key foodborne pathogens and biofilms on common food contact surfaces, including plastic, stainless steel, and rubber. The CQDs exhibited a quasi-spherical shape with particle sizes ranging from 1.7 to 9.0 nm and contained abundant oxygen- and nitrogen-functional groups, as confirmed by FT-IR and XPS analyses. The CQDs showed significant antimicrobial activity, with minimum inhibitory concentrations (MICs) and minimum bactericidal concentrations (MBCs) against *Salmonella typhimurium*, *Escherichia coli* O157: H7, *Listeria monocytogenes*, and *Staphylococcus aureus* of 2200/2800 µg/mL, 1400/2000 µg/mL, 1200/1800 µg/mL, and 400/600 µg/mL, respectively. Time-kill assays confirmed these results. In biofilm tests, *S. typhimurium* formed biofilms more easily than *L. monocytogenes*. Washing with CQD solution for 5 min reduced biofilm presence by 81.6–91.5% for *S. typhimurium* and over 74% for *L. monocytogenes*, with more than 94% reduction after 10 min of treatment (over 94% for *S. typhimurium*; 95.8–98.8% for *L. monocytogenes*) across all surfaces, especially on plastic and stainless steel. These findings indicate that onion peel-derived CQDs are promising, eco-friendly agents for disrupting biofilms and turning undervalued waste into valuable products.

## 1. Introduction

Globally, approximately 1.3 billion tons of food are lost or wasted each year, accounting for nearly one-third of all food produced for human consumption [1]. Among agricultural by-products, onion peels make up a significant portion of waste generated during processing. In countries like the United Kingdom, the Netherlands, and Spain, about 500,000 tons of onion peel waste are produced annually [2]. In the Republic of Korea, onions are among the top 10 most consumed agricultural products, with a production volume of 1,173,000 tons as of 2023; onion peels typically account for approximately 18–26% of the total onion weight [3,4], which is usually discarded. These peels are rich in bioactive compounds, especially flavonoids and phenolics such as quercetin, which have strong antioxidant and antimicrobial activities [5]. Due to these properties, onion peel extracts have been studied for various food applications, including natural preservatives, antioxidant additives, and packaging materials [6,7,8,9].

Biofilms are structured microbial communities embedded in a self-produced, extracellular polymeric substance (EPS) matrix, which allows them to adhere firmly to both biotic and abiotic surfaces [10]. Compared to planktonic cells, biofilm-associated bacteria exhibit increased resistance to environmental stressors, including disinfectants [11]. In food processing facilities, especially poultry plants, pathogens such as *Salmonella* spp. and *Listeria monocytogenes* form persistent biofilms on surfaces such as conveyor belts and stainless-steel equipment, leading to cross-contamination and recurrent outbreaks [12,13]. These biofilms are difficult to detect and remove completely during routine cleaning procedures. Repeated exposure to disinfectant concentrations may even contribute to sublethal injury and antimicrobial resistance, posing a long-term threat to food safety and public health [14,15,16]. Accordingly, ongoing efforts have focused on developing natural antimicrobials, such as essential oils, which are viewed as safer alternatives to synthetic disinfectants [17,18]. However, their low stability and limited efficacy have restricted widespread application [19], highlighting the need for alternative materials with improved performance.

Carbon quantum dots (CQDs) are nanomaterials smaller than 10 nm that have attracted attention because of their biocompatibility, non-toxicity, and tunable surface properties [20]. Bottom-up synthesis methods, such as hydrothermal carbonization, allow the production of CQDs from renewable biomass sources [21,22]. CQDs derived from food waste have been widely studied for their antioxidant and antibacterial properties, and their integration into packaging materials and food additives has proven effective in inhibiting microbial growth and extending the shelf life of meat and tofu products [23,24,25]. Although CQDs have been investigated as washing agents for fresh produce [26], their application for biofilm removal on food contact surfaces has not yet been reported.

Among various biomass sources used for CQD synthesis, onion peels are especially suitable because of their high carbohydrate content (approximately 88.6%) [5], which provides a rich carbon matrix for effective carbonization. This level is considerably higher than that of other common precursors such as banana peels and orange peels [27,28], confirming that onion peels are an efficient and sustainable precursor for CQD production.

Although onion peels have been investigated for their neuroprotective, anticancer, anti-obesity, antidiabetic, and antibacterial properties [29], their potential as a precursor for functional nanomaterials remains largely underexplored. The high carbohydrate and phenolic content of onion peels not only supports their traditional use in extract-based applications but also provides the structural and chemical basis for efficient carbonization into CQDs possessing biologically active surface groups [30,31]. Previous studies have demonstrated that biomass rich in flavonoids and heteroatoms can yield CQDs with enhanced ROS-generating capacity and stronger interactions with microbial membranes, suggesting that onion peels may produce CQDs with superior antimicrobial activity compared with other waste-derived precursors [32]. Furthermore, although CQDs have been incorporated into food packaging materials and evaluated as antimicrobial agents for fresh produce, their use in disrupting established biofilms on food-contact surfaces has not yet been examined. This gap highlights the importance of exploring onion-peel-derived CQDs as a sustainable and potentially more effective sanitizing approach for food-related environments.

Therefore, the objectives of this study were as follows: (1) to synthesize carbon quantum dots from onion peels, (2) to evaluate the antimicrobial and antioxidant properties of the synthesized CQDs, and (3) to assess their effectiveness in removing biofilms from various food contact surfaces, with potential use as a sanitizer. This study is the first to demonstrate the use of onion-peel-derived CQDs as a sanitizer for removing biofilms from various food contact surfaces, representing a significant and innovative contribution to the field.

## 2. Materials and Methods

### 2.1. Synthesis of the Carbon Quantum Dots (CQDs)

Onion peel powder was purchased from a commercial online supplier (Wonju, Republic of Korea). Onion-peel-derived CQDs were synthesized via hydrothermal carbonization following a previously reported method [33]. Onion peel powder (1.5 g) was dispersed in 50 mL of distilled water and stirred with a magnetic stirrer at 200 rpm for 5 min. The suspension was then transferred to a 100 mL Teflon-lined autoclave and heated in a muffle furnace at 200 °C for 7 h. Four muffle furnace chambers were operated simultaneously as one set. After cooling to room temperature for 6 h, the resulting brown solutions were centrifuged at 10,000 rpm for 15 min to remove larger particulate matter. The supernatants were then filtered through syringe-mounted Whatman membrane filters (25 mm diameter, 0.22 µm pore size; Whatman, Little Chalfont, UK) to obtain purified CQDs. Filtrates collected from all four chambers were pooled, and 1 mL of the mixed solution was dried in a Petri dish at 105 °C for 1 h to evaluate batch-to-batch reproducibility and determine the final CQD concentration. The resulting concentration was 6500 µg/mL, with four independent batches yielding comparable values, confirming reproducibility. The prepared CQD solution was stored at 4 °C until further analysis.

### 2.2. Characterization of the Carbon Quantum Dots

The microstructure of the CQDs was examined using a transmission electron microscope (TEM) (FE-TEM, JEM-2100F, JEOL Ltd., Tokyo, Japan) to verify the size and shape of the synthesized CQDs. The average particle size distribution of the CQDs was determined using ImageJ software (ImageJ 1.53k; Wayne Rasband and contributors, National Institutes of Health, Bethesda, MD, USA). The light absorption spectrum of the CQDs was analyzed with a Multiskan GO (Thermo Scientific, Waltham, MA, USA) in the range of 250–700 nm at 25 °C. Fluorescence responses were measured using a fluorescence spectrophotometer (F-7100 FL, Hitachi High-Tech Science Corp., Tokyo, Japan). The synthesized CQDs were characterized with an FT-IR spectrometer (TENSOR 37 spectrophotometer with OPUS 6.0 software, Billerica, MA, USA) over the range of 4000–500 cm^−1^ to generate a Fourier transform infrared (FT-IR) spectrum, which was used to identify functional groups and elemental composition. X-ray photoelectron spectroscopy (XPS) analysis was performed with a K-alpha X-ray photoelectron spectrometer (Thermo Scientific, Waltham, MA, USA). Additionally, a zeta potential analyzer (Zetasizer Nano-ZS-ZEN, Malvern Instruments Ltd., Malvern, UK) was used to evaluate the surface charge of the CQDs.

### 2.3. Antimicrobial Activity of the Carbon Quantum Dots

#### 2.3.1. Minimum Inhibitory Concentrations (MICs) and Minimum Bactericidal Concentrations (MBCs) of the CQDs

Test microbial strains, including *Salmonella typhimurium* (ATCC 13311), *Escherichia coli* O157: H7 (NCTC 12079), *L. monocytogenes* (KCCM 43155), and *Staphylococcus aureus* (ATCC 13565), were obtained from the Korean Culture Center of Microorganisms (KCCM, Seoul, Republic of Korea). To evaluate the antibacterial activity of CQDs against these four bacterial strains, MICs and MBCs were determined using the following method [34]. Using a 96-well plate, 50 µL of prepared bacterial culture (4.5–5.5 log CFU/mL) of each strain was inoculated into 100 µL of various final concentrations of CQD solution (400, 600, 800, 1000, 1200, 1400, 1600, 1800, 2000, 2200, 2400, 2600, 2800, and 3000 µg/mL), along with either 50 µL of tryptic soy broth (TSB, KisanBio., Ltd., Seoul, Republic of Korea; for *S. aureus*, *L. monocytogenes*, and *E. coli* O157: H7) or 50 µL of brain–heart infusion (BHI, Oxoid, Hampshire, England; for *S. typhimurium*). In the control group, the bacterial inoculum was replaced with an equal volume of 0.1% peptone water. The 96-well plates were incubated at 36 °C for 24 h (VS-1203P3V, Vision Scientific Co., Ltd., Daejeon, Republic of Korea), and the MIC was subsequently determined by measuring the optical density (OD) at 600 nm using a Multiskan GO (Thermo Scientific, Waltham, MA, USA). To determine the MBC, the entire solution from each well was plated onto tryptic soy agar (TSA, Oxoid, Hampshire, UK) for *S. aureus* and *S. typhimurium*, nutrient agar (NA, KisanBio., Ltd., Seoul, Republic of Korea) for *E. coli* O157: H7, or TSA supplemented with 0.6% yeast extract for *L. monocytogenes*. MIC was defined as the lowest concentration that resulted in an OD value identical to that of the control. The MBC was defined as the lowest concentration at which no bacterial colonies were observed on the corresponding agar plates.

#### 2.3.2. Time-Kill Assay

For the time-kill assay of CQDs against the four bacterial strains, 2 mL of each bacterial culture (4.5–5.5 log CFU/mL) was inoculated into 11.4 mL of TSB for *S. aureus, L. monocytogenes*, and *E. coli* O157: H7 or BHI for *S. typhimurium*. BHI broth was used for *S. typhimurium* because it is commonly employed for its cultivation and serves as an effective non-selective enrichment medium. For both TSB and BHI, 6.6 mL of either 0.1% peptone water (control) or CQD solution (1600 µg/mL) was added. The inoculated solutions were incubated at 36 °C for 24 h with shaking at 140 rpm using a rotary shaker (VS-8480SR, Vision Scientific Co., Ltd., Daejeon, Republic of Korea). The concentration of 1600 µg/mL was selected because, based on the MIC/MBC results for the four tested strains, it provided an appropriate level at which strain-dependent differences in antimicrobial susceptibility could be clearly distinguished.

Samples were sterilely collected and serially diluted at each time point, then plated onto TSA for *S. aureus* and *S. typhimurium*, NA for *E. coli* O157: H7, or TSA supplemented with 0.6% yeast extract for *L. monocytogenes*. This was done at each time point (0, 3, 6, 9, and 12 h post-inoculation). The plates were then incubated at 36 °C for 24 h. The behavior of each bacterium treated with CQDs was determined by assessing the changes in colony counts at each time point.

### 2.4. Antioxidant Activity of the Carbon Quantum Dots

The antioxidant activity of the CQDs was assessed using the ABTS assay [35] at concentrations of 12.5, 25, 50, 75, and 100 µg/mL. Because the onion-peel–derived CQDs are hydrophilic, the ABTS assay, which functions effectively in both aqueous and organic systems, was selected as the appropriate method for evaluating their antioxidant capacity.

A predefined volume of CQD solutions was mixed with 5 mL of ABTS solutions and left at room temperature for 10 min. The absorbance was then measured at 734 nm using a UV-Vis spectrophotometer (Mecasys Optizen POP Series, Seoul, Republic of Korea), and the antioxidant activity was evaluated, as described below:

$$\mathrm{Antioxidant}\;\mathrm{activity}\;\left(\%\right)=\;\frac{A_{0}-A_{T}}{A_{0}}\times 100$$

where A_0_ and A_T_ represent the absorbance values of the control and CQD samples. Vitamin C was included as a positive control, and its antioxidant activity was assessed using the same procedure.

### 2.5. Analysis of Biofilm Formation on Food Contact Surfaces

#### 2.5.1. Preparation of Strains for Biofilm

The bacterial strains used for biofilm formation were *S. typhimurium* (ATCC 13311) and *L. monocytogenes* (KCCM 43155). Each strain was stored in sterilized BHI (*S. typhimurium*) or TSB (*L. monocytogenes*) supplemented with 20% glycerol at −80 °C. For experimental use, 10 µL of each frozen stock was inoculated into 10 mL of fresh BHI or TSB and incubated at 36 °C for 24 h using a rotary shaker.

#### 2.5.2. Biofilm Formation

Biofilm was formed on food contact surfaces using the following method [36]. Three surface materials commonly used in food processing environments (plastic, stainless steel, and rubber) were purchased from an online market. Each material was cut into coupons measuring 3 cm × 2 cm. All coupons were initially cleaned with 70% ethanol to remove surface contaminants. The stainless steel and plastic coupons were sterilized by autoclaving at 121 °C for 15 min. The rubber coupons were immersed in 70% ethanol for 30 min, dried at 80 °C for 20 min, and subsequently exposed to UV light (G40T10, Sankyo Denki, Tokyo, Japan) for 10 min inside a clean bench (CB200B, Vision Scientific Co., Ltd., Daejeon, Republic of Korea) prior to use. Sterile coupons of each material were immersed in 25 mL BHI for *S. typhimurium* or 25 mL of TSB for *L. monocytogenes* in 50 mL conical tubes, in which one milliliter of standardized bacterial culture (10^6^ CFU/mL) was inoculated. The inoculated tubes were incubated at 36 °C for 3 days to allow biofilm formation on the coupon surfaces. After incubation, loosely attached cells were gently removed by rinsing each coupon with sterile distilled water.

### 2.6. Assessing the Sanitizing Efficiency of Carbon Quantum Dots on Food Contact Surfaces

**Figure 1:**
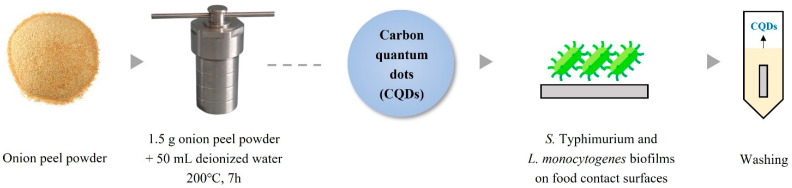
Overview of the preparation and application of onion-peel-derived carbon quantum dots as a sanitizer for biofilm removal.

To assess the cleaning effectiveness of CQDs as a sanitizer, the coupons were immersed in 50 mL conical tubes containing 30 mL of undiluted CQD solution (6500 µg/mL) for 5, 10, and 15 min (see Figure 1). After treatment, the coupons were rinsed again with sterile distilled water to remove any remaining CQDs.

To evaluate the extent of biofilm formation and the cleaning effectiveness of CQDs, each coupon was tested using a modified beads assay [37]. Biofilm-covered coupons were placed into 50 mL conical tubes with 12 mL of sterile peptone water and three sterile glass beads, then vigorously vortexed for 2 min to detach the attached cells. The resulting suspension was either directly plated or serially diluted. For quantification, *S. typhimurium* was plated on TSA, while *L. monocytogenes* was plated on TSA supplemented with 0.6% yeast extract. The plates were incubated at 36 °C for 24 h, after which colonies were counted and expressed as log CFU per coupon. To assess the cleaning efficacy of CQDs, the percent reduction in biofilm cells was calculated using the following equation:

$$\mathrm{Reduction}\;\left(\%\right)=\left[1-10^{-(\log N_{0}-\log N_{T})}\right]\times 100$$

where $N_{0}$ is the initial microbial count on the coupon (log CFU/coupon) before treatment and $N_{T}$ is the microbial count (log CFU/coupon) after CQD treatment.

### 2.7. Statistical Analysis

All experiments were performed in triplicate. One-way analysis of variance (ANOVA) was used, and significant differences among treatments were identified using Duncan’s multiple range test. Differences between the two groups were assessed with a *t*-test. Normality was checked with the Shapiro–Wilk test, and homogeneity of variance was verified using Levene’s test. Statistical analyses were performed with SAS software (version 9.3; SAS Institute Inc., Cary, NC, USA).

## 3. Results and Discussion

### 3.1. Morphology of the Onion-Peel Derived Carbon Quantum Dots

**Figure 2:**
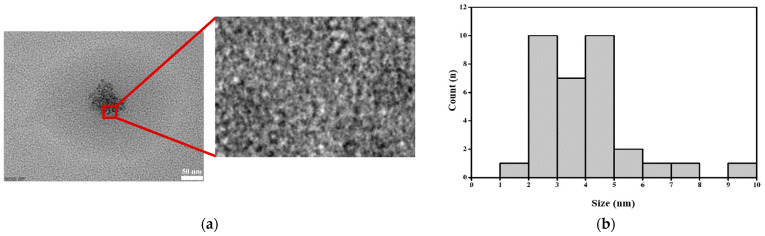
Characterization of the CQDs: (**a**) TEM images and (**b**) size distribution of the CQDs.

The microstructure and distribution of the particle sizes of the onion-peel-derived CQDs are shown in Figure 2a,b. The CQDs exhibited a quasi-spherical shape, ranging from 1.7 to 9.0 nm, with an average diameter of 3.9 nm. In previous work, similar CQDs had an average size of approximately 5 nm [38]. The average particle size of the CQDs derived from onion peels in this study was smaller compared to other reported CQDs synthesized from different food waste sources, such as tangerine peels (8.2 ± 0.3 nm), coffee grounds (5.8 ± 0.1 nm, 6.4 ± 0.9 nm, and 6.2 ± 0.8 nm for raw, roasted, and spent, respectively), and potato skins (9.3 ± 0.2 nm) [23,39,40]. Because smaller nanoparticles typically provide a larger surface area for interaction with bacterial cells [41], the smaller size of the onion-peel-derived CQDs in this study may enhance their antimicrobial activity.

### 3.2. Optical Properties of Onion-Peel-Derived Carbon Quantum Dots

**Figure 3:**
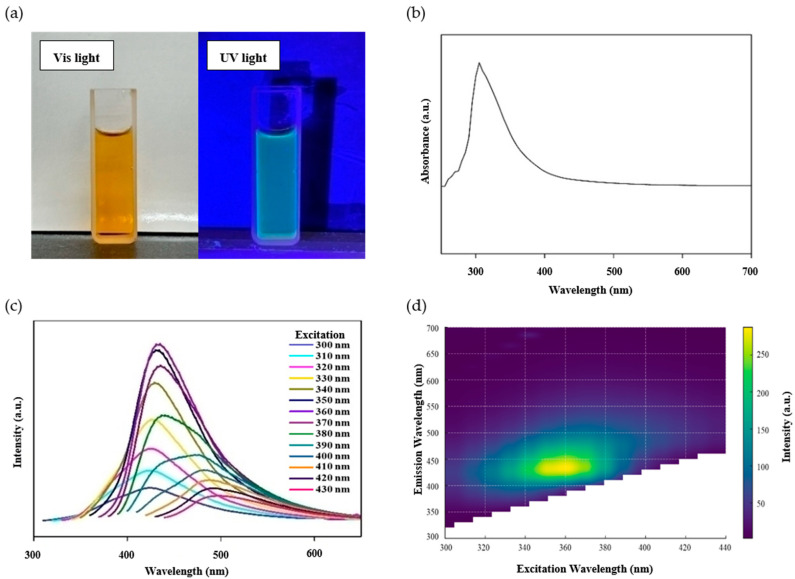
Characterization of the CQDs: (**a**) CQD solution under visible and UV light, (**b**) UV-Vis spectrum, (**c**) FL emission spectra, and (**d**) heat map of the CQDs.

The CQD suspension appeared brown under visible light, whereas it exhibited bright blue fluorescence under 360 nm UV illumination (Figure 3a), consistent with the quantum confinement effect [42]. These observations confirm the successful formation of CQDs from onion peel via the hydrothermal process.

The UV-Vis absorption spectrum of the CQDs displayed distinct peaks in the UV region between 300 and 340 nm (Figure 3b). These peaks are due to electronic transitions, such as π–π* (C=C bond) or n–π* transition (C=O), and align with previously reported optical patterns of similar carbon-based nanomaterials [43,44]. Furthermore, Figure 3c,d show the fluorescence emission spectra of the CQDs under excitation wavelengths ranging from 300 to 430 nm. The CQDs exhibited clear excitation-dependent emission, with the emission peak shifting to longer wavelengths as the excitation wavelength increased. The strongest fluorescence was observed at the optimal excitation wavelength of 360 nm, whereas further increasing the excitation wavelength to 430 nm resulted in reduced emission intensity. These optical characteristics confirm the successful synthesis of CQDs from onion peel.

### 3.3. FT-IR and XPS Analysis

**Figure 4:**
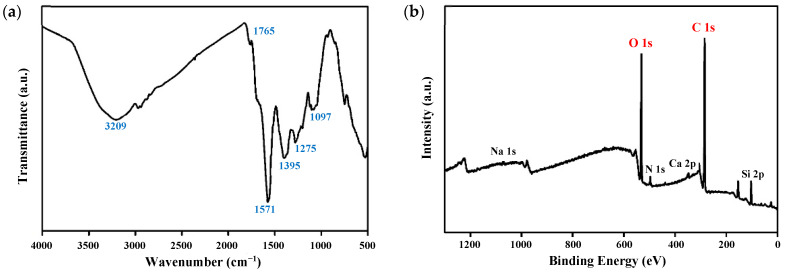
Characterization of the CQDs with (**a**) an FT-IR spectrum and (**b**) an XPS spectrum.

The chemical structure and composition of the CQDs were also examined using FT-IR (Figure 4a) and XPS (Figure 4b). The FT-IR spectrum displayed characteristic absorption bands at around 3209 cm^−1^, 1765 cm^−1^, and 1571 cm^−1^, which correspond to O–H/N–H stretching vibrations, carbonyl (C=O) stretching, and C=C stretching of polycyclic aromatic hydrocarbons, respectively [45]. Additional peaks at 1395 cm^−1^, 1275 cm^−1^, and 1097 cm^−1^ were linked to the symmetric stretching vibrations of C–N (amide group), O–H bending (phenolic hydroxyl groups), and C–O–C bonds, respectively [46]. These findings confirm that the onion-peel CQDs contain C=O at 1765 cm^−1^, O–H/N–H at 3209 cm^−1^, and C–N amide groups at 1395 cm^−1^, which are related to water-soluble and various oxygen- and nitrogen-containing functional groups, indicating potential antimicrobial activity on the CQD surface. These results align with the UV-Vis absorption spectrum shown in Figure 3b.

The chemical composition and surface elements of the CQDs showed key peaks of Na, O, N, Ca, C, and Si, as illustrated in Figure 4b. These peaks corresponded to binding energies at 1071, 532, 490, 306, 285, and 105 eV, respectively [47,48,49]. Surface chemical elements such as Na, N, and Ca are likely to enhance antimicrobial activity by disrupting bacterial cell walls [34]. The detection of Si on the surface of the CQDs derived from onion peels indicated high silicon content in the plant cell walls [50]. XPS analysis identified elements like Na and Ca, which are usually electropositive [51,52], along with N and O, commonly involved in electronegative surface functionalities [53,54]. Carbon was also present and probably forms the backbone of oxygen-containing groups such as hydroxyl, carbonyl, and carboxyl groups [55]. The strong C and O peaks in the XPS spectrum support the prevalence of these functional groups, which may contribute to a negative surface charge [56,57]. Additionally, nitrogen’s presence suggests possible participation of reactive nitrogen species (RNS), potentially boosting the antimicrobial effects of the CQDs [58].

The functional groups identified by FT-IR (C–N, C=O, C–O–C; Figure 4a) and the elemental signals detected by XPS (O 1s, C 1s, Na 1s, and Ca 2p; Figure 4b) contribute to the overall surface chemistry of the CQDs, resulting in a net negative surface charge. Surface charge has been widely recognized as a key determinant of the antimicrobial activity of carbon-based nanomaterials, as reported in previous studies [21,23]. For instance, CQDs derived from tangerine peel exhibited dominant O 1s and C 1s compositions and a negative charge, which enhanced antimicrobial efficacy against the Gram-positive bacterium *Bacillus cereus* through charge-mediated interactions with the cell wall [23]. Similarly, negatively charged CQDs synthesized from various coffee beans displayed stronger antimicrobial activity against Gram-positive (*S. aureus*, *L. monocytogenes*) than Gram-negative (*E. coli*, *S. enterica*) bacteria [39]. Based on these findings, the negatively charged CQDs prepared from onion peel powder in the present study were expected to exhibit substantial antimicrobial activity.

According to the zeta potential analysis, the onion-peel CQDs showed a negative surface charge (−6.3 mV). This finding aligns with the presence of hydroxyl and carbonyl functional groups identified in the FT-IR and XPS analyses.

### 3.4. Antimicrobial Activity of Onion-Peel-Derived Carbon Quantum Dots

The antibacterial activity of the CQDs against *S. typhimurium*, *E. coli* O157: H7, *L. monocytogenes*, and *S. aureus* was assessed using MIC and MBC tests. The MIC/MBC values for *S. typhimurium*, *E. coli* O157: H7, *L. monocytogenes*, and *S. aureus* were 2200/2800 µg/mL, 1400/2000 µg/mL, 1200/1800 µg/mL, and 400/600 µg/mL, respectively. These findings show that CQDs have the strongest antibacterial effect against *S. aureus*.

**Figure 5:**
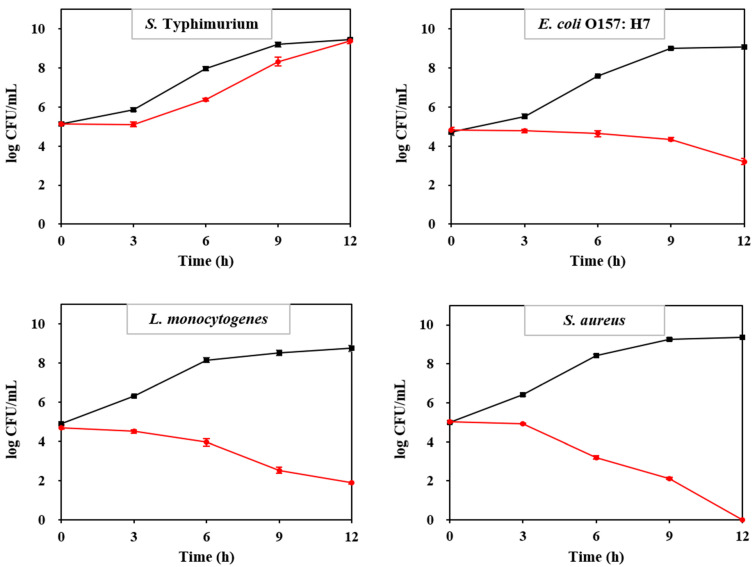
Time-kill assay of CQDs in broth against *S. typhimurium*, *E. coli* O157: H7, *L. monocytogenes*, and *S. aureus*. Control (■, black lines); CQD-treated group (●, red lines).

Figure 5 presents the results of the time-kill assay illustrating the bactericidal effect of the CQDs. Compared to the control, the growth of *S. typhimurium* was slower in broth containing 1600 µg/mL of CQDs. Conversely, both *E. coli* O157: H7 and *L. monocytogenes* growth was significantly inhibited over time in broth containing 1600 µg/mL of CQD. Notably, *S. aureus* was eliminated within 12 h in broth containing 1600 µg/mL of CQDs.

These observations were consistent with the MIC and MBC results, indicating that Gram-positive bacteria were more susceptible to onion-peel-derived CQDs than Gram-negative bacteria. As noted above, this effect can be attributed to the functional groups present on the CQDs, such as hydroxyl and carbonyl groups identified through FT-IR and XPS analyses, as well as their negative surface charge confirmed by the zeta potential analysis.

Although the exact antibacterial mechanism of CQDs has not yet been fully clarified, several hypotheses have been proposed in previous studies [46,59,60]. One involves ionic interactions between CQDs and bacterial cell surfaces, which may disrupt membrane integrity. This charge-related interaction could be stronger in Gram-positive bacteria, because their cell walls contain negatively charged peptidoglycan and teichoic acids, while the highly negative lipopolysaccharide (LPS) layer of Gram-negative bacteria might limit such interactions [60,61]. These structural differences may partly explain why greater antibacterial effectiveness was seen against Gram-positive bacteria in this study.

Another proposed mechanism is the generation of reactive oxygen species (ROS) by oxygen-containing functional groups on the surface of CQDs, which can cause oxidative damage and weaken cell wall integrity [45]. Recent evidence now offers more direct mechanistic support for this ROS-mediated pathway. Xia et al. (2025) [62] demonstrated that N-CQDs caused significant intracellular ROS buildup in methicillin-resistant *S. aureus* and *S. aureus* with fluorescence intensities similar to those seen in H_2_O_2_-treated positive controls. Furthermore, SEM images showed progressive deformation and membrane rupture as N-CQD concentrations increased, while TEM analysis confirmed the complete loss of membrane integrity in treated bacterial cells. These results suggest that ROS-driven oxidative stress and membrane disruption are key antimicrobial mechanisms of CQDs.

Additionally, the nanoscale size of CQDs may allow quick penetration into bacterial cells, disrupting essential metabolic processes [59]. Overall, these findings indicate that onion-peel-derived CQDs combat bacteria through multiple complementary pathways, highlighting their strong potential as effective antimicrobial agents in various food industry applications. However, the exact antimicrobial mechanisms of CQDs still need further research. Future studies will compare intracellular ROS production and cytoplasmic membrane permeability among CQDs made from different precursor materials to better understand their modes of action.

### 3.5. Antioxidant Activity of Onion-Peel-Derived Carbon Quantum Dots

**Figure 6:**
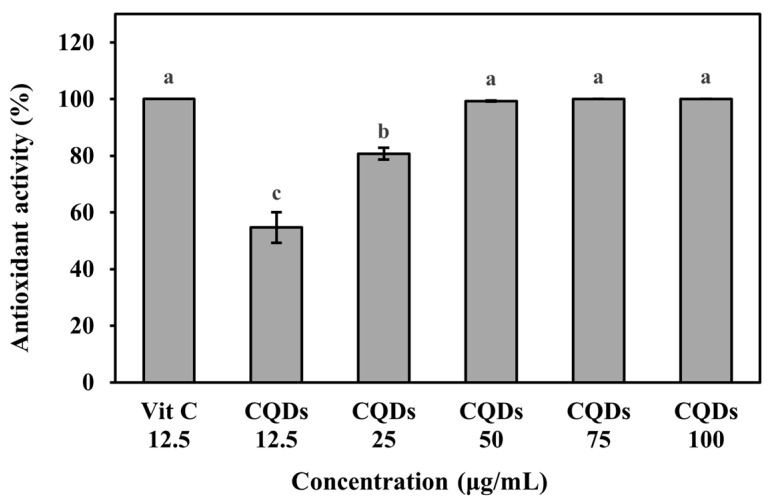
Antioxidant activity of CQDs measured by ABTS. a–c values of each treatment with different letters indicate a significant difference (*p* < 0.05).

The antioxidant activity of the CQDs was evaluated using the ABTS assay, and the results are presented in Figure 6. As the concentration of CQDs increased, a significant enhancement in radical scavenging activity was observed (*p* < 0.05). The positive control, vitamin C, exhibited 100% scavenging activity at a concentration of 12.5 µg/mL, whereas 50% radical scavenging activity was observed at a concentration of 12.5 µg/mL of CQDs and 100% scavenging activity of CQDs was observed at concentrations of 50 µg/mL and above. For comparison, CQDs synthesized from banana peels demonstrated 72.4% ABTS-radical scavenging activity at 50 µg/mL, which increased to 99% at 75 µg/mL [25]. Similarly, eggplant-peel-derived CQDs exhibited a concentration-dependent increase in antioxidant activity, reaching nearly 100% at 100 µg/mL [63]. In addition, coffee-ground–derived CDs achieved 100% ABTS-radical scavenging activity at 75 µg/mL [39], indicating that onion-peel–derived CQDs attain comparable maximal activity at a lower concentration. Collectively, these results suggest that CQDs synthesized from discarded onion peels possess strong, dose-dependent antioxidant capacity and offer added value as a natural antioxidant agent for food applications.

The antioxidant capacity of CQDs is largely attributed to oxygen-containing functional groups on their surface, which can donate hydrogen atoms (H^•^) from hydroxyl groups to ABTS^•+^, reducing and stabilizing the radical species [64]. This reaction is generally understood to involve a combination of hydrogen donation, electron transfer, and radical addition mechanisms. The CQDs exhibited higher antioxidant activity in the ABTS assay than in the DPPH assay, because their hydrophilic nature allows more efficient interaction and solubilization in the aqueous ABTS reagent compared with the methanol-based DPPH solution [25].

### 3.6. Biofilm Formation on Food Contact Surfaces

**Figure 7:**
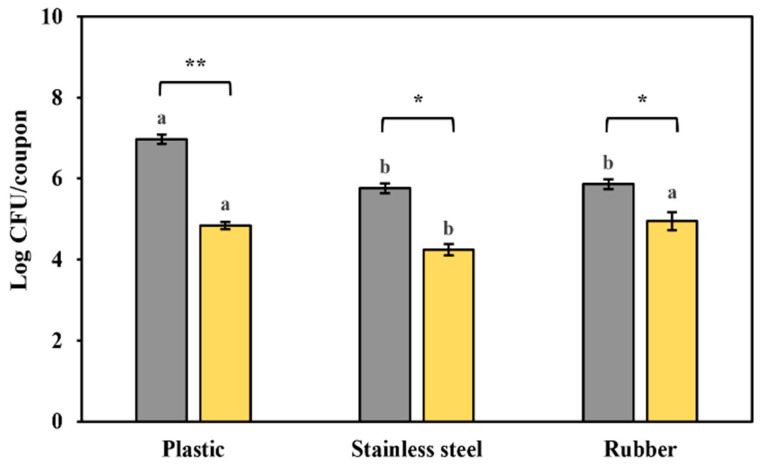
Biofilm formation of *S. typhimurium* and *L. monocytogenes* on plastic, stainless steel, and rubber. Different lowercase letters between surface types for each pathogen indicate a significant difference (*p* < 0.05). There is a significant difference in biofilm formation ability between *S. typhimurium* and *L. monocytogenes* on each surface by the *t*-test (* *p* < 0.05, ** *p* < 0.01). *S. typhimurium* (■); *L. monocytogenes* (■).

The biofilm-forming capabilities of *S. typhimurium* and *L. monocytogenes* on plastic, rubber, and stainless steel are shown in Figure 7. Under the same incubation conditions at 36 °C for 72 h, *S. typhimurium* produced significantly more biofilm on all tested surfaces than *L. monocytogenes*. This matches previous findings that Gram-negative bacteria like *S. typhimurium* are better at forming biofilms on surfaces compared to Gram-positive bacteria such as *L. monocytogenes* [65,66]. *S. typhimurium* forms more biofilm than *L. monocytogenes* on plastic surfaces [67]. These results further support the current findings.

Among the surfaces tested, *S. typhimurium* produced the highest level of biofilm on plastic, followed by stainless steel and rubber, where no significant difference in biofilm levels was observed. In contrast, *L. monocytogenes* formed similar levels of biofilm on both plastic and rubber, with a significantly lower amount on stainless steel. Stainless steel is generally classified as a hydrophilic material, while plastic and rubber are considered hydrophobic [68,69]. Microorganisms tend to adhere more readily to hydrophobic surfaces [68,69,70], which aligns with the trends seen in this study. These results suggest that biofilm formation varies depending on the bacterial species, and the type of food contact surface plays a key role in biofilm development. Therefore, effective cleaning and disinfection of biofilms on food contact surfaces in food processing environments are essential to prevent cross-contamination of microorganisms and ensure food safety.

### 3.7. Sanitizing Efficacy of Carbon Quantum Dots on Biofilm Removal

**Figure 8:**
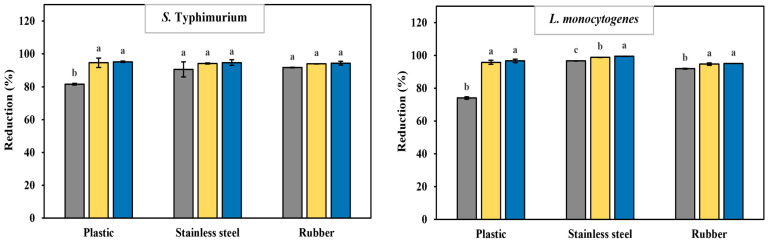
The effect of the CQD solution on biofilm reduction in *S. typhimurium* and *L. monocytogenes* according to the kind of food contact surface and washing time. Different lowercase letters between treatment times indicate a significant difference (*p* < 0.05). Gray, yellow, and blue bars indicate biofilm reduction after 5, 10, and 15 min of washing, respectively. Reduction (%) after 5 min (■), 10 min (■), and 15 min (■).

In this study, the synthesized and undiluted CQD solution was used for biofilm removal (6500 μg/mL). Although the MBC values for *S. typhimurium* and *L. monocytogenes* planktonic cells are 2800 and 1800 μg/mL, respectively, biofilms are known to be 10–1000 times more resistant to antimicrobials, including common sanitizers such as chlorine, peracetic acid, and quaternary ammonium compounds [71,72]. The effectiveness of the CQDs in removing biofilms was tested on *S. typhimurium* and *L. monocytogenes* biofilms formed on three food-contact surface coupons (Figure 8).

After 5 min of treatment in 30 mL of undiluted CQD solution (6500 µg/mL), the reductions (%) in *S. typhimurium* biofilm on plastic, stainless steel, and rubber surfaces were 81.6%, 90.1%, and 91.5%, respectively. The greatest reduction in *S. typhimurium* biofilm was observed on rubber. However, when the washing time was increased to 10 min, the reductions improved to 94.3%, 94.2%, and 94.1%, respectively. A statistically significant increase in cleaning effectiveness was observed only on the plastic surface between the 5 min and 10 min treatments, and no further reduction in biofilm cells occurred with the 15 min treatment. For *L. monocytogenes*, the 5 min treatment resulted in reductions of 74.0%, 96.7%, and 92.8% on plastic, stainless steel, and rubber, respectively. When the washing time was increased to 10 min, the reductions improved to 95.8%, 98.8%, and 95.3%, respectively, with statistically significant differences seen on all three surfaces compared to the 5 min treatment. The greatest reduction was observed in *L. monocytogenes* biofilm on the stainless-steel coupon.

When comparing the biofilm reduction after a 10 min washing treatment, *L. monocytogenes* showed greater biofilm removal than *S. typhimurium* on all tested surfaces. This trend may be due to *L. monocytogenes* having a weaker biofilm-forming ability than *S. typhimurium*, as observed in this study, which makes it more vulnerable to the CQD washing treatment. Additionally, the differences in biofilm removal could also be related to variations in EPS composition between the two bacterial strains. Although both pathogens produce EPS containing polysaccharides, proteins, and eDNA, structural and charge differences may result from specific components such as teichoic acids in Gram-positive bacteria and lipopolysaccharides (LPS) in Gram-negative bacteria [73,74,75]. Given the negatively charged nature of onion-peel-derived CQDs, stronger interactions with positively charged elements in the EPS may have enhanced the disruption and removal of *L. monocytogenes*. When the washing time was extended to 15 min, no significant reduction in biofilm was seen for either bacterial strain, except for *L. monocytogenes* on the stainless-steel surface, where further reduction was observed. Considering both effectiveness and practicality, a 10 min washing period seems to be the best condition for biofilm removal using the synthesized CQD solution from onion peels.

Previous studies have extensively examined how effective various sanitizers are at removing biofilms from food contact surfaces [76,77,78]. The success of sanitizing treatments varies greatly depending on the microorganism type and the sanitizer used. For example, treatment with 100 ppm chlorine eliminated *Salmonella* Weltevreden biofilms on plastic, cement, and stainless-steel surfaces after 20, 20, and 15 min of exposure, respectively [79]. Additionally, peracetic acid and sodium hypochlorite reduced *S. aureus* biofilms by 2.0–3.3 and 1.5–2.1 log CFU/cm^2^, respectively [80]. NaClO treatment for 5 min resulted in 93.9%, 88.3%, 81.4%, and 41.1% reductions in *Listeria innocua* biofilms on polypropylene, stainless steel, rubber, and glass, respectively. Under the same treatment duration, 120 ppm SAEW achieved a 97.3% reduction in *L. innocua* biofilms on polypropylene, showing the highest efficacy among the tested surfaces [36]. In this study, the washing solution containing onion-peel-derived CQDs achieved up to a 98.8% reduction in *L. monocytogenes* biofilms on stainless steel within 10–15 min. This suggests that the biofilm reduction effectiveness of onion-peel-derived CQDs is comparable to or greater than that reported for conventional sanitizers in previous research [36,76,79]. These results reaffirm that the effectiveness of sanitizing treatments can vary widely depending on the microorganism type and sanitizer used. Our findings indicate that onion-peel-derived CQDs are effective at removing biofilms from food contact surfaces and show promise as a cleaning agent. Since few studies have directly compared the susceptibility of biofilms with different structural features to antimicrobial treatments, further research is necessary.

In food processing environments, various bacterial species often form complex multispecies biofilms [16]. Based on this study’s findings, further evaluation of CQDs’ effectiveness against multispecies biofilms would offer valuable insights into their practical use. Additional research is also necessary to assess the cytocompatibility of CQDs and to confirm their effectiveness and scalability in real-world or industrial settings.

Recent studies have identified CQDs in various heat-processed foods, including coffee, cola, grilled meat, smoked fish, grilled chicken, and fried hamburger patties [81]. This evidence indicates that humans have been unknowingly ingesting naturally occurring CQDs for centuries, well before the development of engineered nanomaterials. Because of their natural origin, biodegradability, and proven functional properties, CQDs hold great promise for future applications across multiple sectors.

## 4. Conclusions

Carbon quantum dots (CQDs) were successfully produced from discarded onion peels using a simple hydrothermal method. Their potential as natural antioxidants and sanitizers for removing biofilms on food contact surfaces such as plastic, stainless steel, and rubber was evaluated. The CQDs exhibited clear, dose-dependent antimicrobial effects against four major foodborne pathogens, with *S. aureus* being the most susceptible, followed by *L. monocytogenes*, *E. coli* O157: H7, and *S. typhimurium*. Overall, the CQDs proved more effective against Gram-positive bacteria. Biofilm tests showed that *S. typhimurium* formed stronger biofilms than *L. monocytogenes* on all surfaces, indicating species-specific differences. Washing with CQDs significantly reduced biofilms over time, with optimal removal after 10 min of treatment for both *L. monocytogenes* and *S. typhimurium* on all tested materials. Due to the simple, scalable production process, low cost, and utilization of food waste as raw material, onion-peel-derived CQDs hold great promise as natural antioxidant and antimicrobial solutions for enhancing food safety and sanitation in food manufacturing environments.

## Data Availability

The original contributions presented in this study are included in the article. Further inquiries can be directed to the corresponding author.
